# Amyloid Proteins and Their Role in Multiple Sclerosis. Considerations in the Use of Amyloid-PET Imaging

**DOI:** 10.3389/fneur.2016.00053

**Published:** 2016-03-31

**Authors:** Jordi A. Matías-Guiu, Celia Oreja-Guevara, María Nieves Cabrera-Martín, Teresa Moreno-Ramos, José Luis Carreras, Jorge Matías-Guiu

**Affiliations:** ^1^Department of Neurology, Hospital Clínico San Carlos, San Carlos Institute for Health Research (IdISSC), Complutense University of Madrid, Madrid, Spain; ^2^Department of Nuclear Medicine, Hospital Clínico San Carlos, San Carlos Institute for Health Research (IdISSC), Complutense University of Madrid, Madrid, Spain

**Keywords:** multiple sclerosis, amyloid PET, biomarkers, white matter, amyloid precursor protein, amyloid, myelin basic protein, positron emission tomography

## Abstract

Thioflavin T derivatives are used in positron-emission tomography (PET) studies to detect amyloid protein deposits in patients with Alzheimer disease. These tracers bind extensively to white matter, which suggests that they may be useful in studies of multiple sclerosis (MS), and that proteins resulting from proteolytic processing of the amyloid precursor protein (APP) may contribute to MS. This article reviews data from both clinical and preclinical studies addressing the role of these proteins, whether they are detected in CSF studies or using PET imaging. APP is widely expressed in demyelinated axons and may have a protective effect in MS and in experimental allergic encephalomyelitis in animals. Several mechanisms associated with this increased expression may affect the degree of remyelination in MS. Amyloid-PET imaging may help determine the degree of demyelination and provide information on the molecular changes linked to APP proteolytic processing experienced by patients with MS.

## Background

Multiple sclerosis (MS) is an autoimmune inflammatory disease of the central nervous system (CNS) that causes inflammatory lesions in the brain and spinal cord and ruptures the blood–brain barrier, leading to demyelination and axonal damage. In normal practice, MS is diagnosed based on clinical symptoms, exclusion of other causes, and findings in cerebrospinal fluid (CSF) and magnetic resonance imaging studies. From a pathogenic point of view, MS is characterized by demyelination, which is attributed to inflammatory mechanisms and followed by neurodegeneration. In most cases, the disease initially presents a relapsing-remitting pattern (RRMS). Patients with this type of MS experience relapses followed by periods of partial or total recovery associated with incomplete remyelination. Remyelinating capacity decreases with time, especially in the secondary progressive form of the disease (1, 2).

Although β-amyloid protein (Aβ) is mainly linked to Alzheimer disease (AD), recent review articles suggest a connection between Aβ and MS (3, 4). One reason that led researchers to associate Aβ with MS was that white matter exhibits significant uptake of the PET tracers binding to this protein (5, 6), whereas white matter lesions associated with AD display lower uptake (7, 8). Myelin loss and breakdown of myelin basic protein (MBP) in AD patients and animal models of AD are associated with aging, the ApoE4 allele, or head injury, all of which are risk factors for AD, as well as with increases in Aβ peptides (9). Several pathology studies of AD have found decreased expression of MBP in the areas presenting Aβ deposition, and decreased Aβ deposition in white matter areas exhibiting greater expression of MBP. MBP has not been detected in amyloid plaques in AD patients (10, 11).

## Amyloid-PET in MS

Positron-emission tomography using different amyloid tracers [Pittsburgh Compound-B (PiB), florbetapir, florbetaben, flutemetamol, and others under study] can detect fibrillar Aβ deposits with high sensitivity and specificity; fibrillar Aβ is therefore considered a biomarker for AD along with levels of Aβ in CSF. This technique enables an *in vivo* pathological and molecular diagnosis, and it is currently included in clinical trial protocols for early detection of AD. Amyloid-PET findings have been proven to correlate well with fibrillar Aβ in neuropathology studies (12). Assessing amyloid tracer uptake in gray matter is a technique for diagnosing AD and for differential diagnosis of neurodegenerative cognitive disorders. Most studies using amyloid-PET aim to assess this imaging technique’s utility for confirming AD diagnosis and predicting progression of mild cognitive impairment to dementia (13, 14). It is also used to diagnose other pathologies presenting with cognitive impairment and which are not linked to Aβ exclusively (15–17). However, changes in amyloid-PET images may also be indicative of other neurological diseases (18). These tracers are thioflavin T derivatives and have been proven more specific than previous compounds based on Congo red and whose chemical basis was the styrylbenzene molecule or Chrysamine G, a derivative of Congo red (19). Thioflavin T analogs bind to amyloid fibrils, unlike Congo red derivatives, which also bind to tau fibrils. Several molecules have been developed by modifying the original structure, giving rise to other tracers that may have different affinities for certain tissues (20–23). Other molecules now being developed may have an even greater affinity for yelin (24).

Molecules currently in use derive from Pittsburgh Compound-A (25), an alternative name for BTA-1 (26), which resulted in PiB. This compound was used to develop three different radioligands: (1) SB1, which gave rise to ^18^F-florbetaben (AV1) and subsequently ^18^F-florbetapir (AV45); (2) ^18^F-flutemetamol; and (3) AZD2184, and subsequently AZD4694 (renamed NAV4694). At present, PiB, florbetaben, florbetapir, and flutemetamol have been tested in clinical trials, and the last three tracers are approved and available for clinical use.

**Figure 1:**
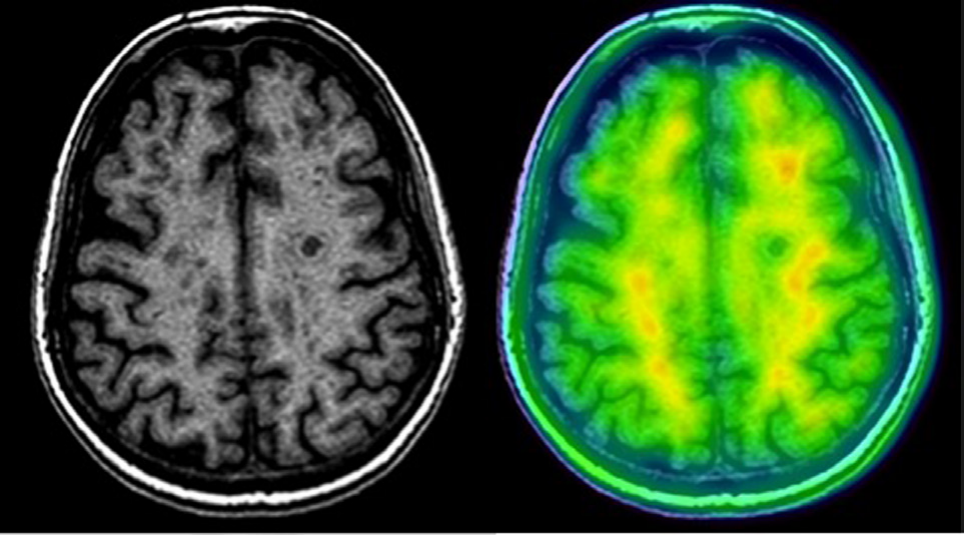
**Amyloid-PET and MRI image of a patient with RRMS using ^18^F-florbetaben**. Note the decreased uptake of the tracer in white matter lesions.

Amyloid tracers detect decreased activity in black hole areas in T1-weighted MR images (27) and in white matter lesions in T2-weighted MR images (28, 29), in both the relapsing-remitting and the progressive forms of MS (Tables 1 and 2; Figure 1). These results showed that amyloid tracers bind extensively to white matter and that uptake decreases with demyelination. This inevitably leads us to question whether the usefulness of amyloid tracers in MS is due to their non-specific binding to white matter, or whether there may be a connection between Aβ and myelination.

**Table 1:** **Studies of amyloid-related measurements in MS**.

Reference	Participants	Measures	Significant findings
(30)	23 cases (14 definite MS, 9 CIS) 40 controls	CSF Aβ Aβ_1–42_	Increased Aβ
(31)	21 cases (CIS) 21 controls	CSF Aβ Aβ_1–40_ Aβ_1–42_	No differences in Aβ
(32)	100 cases MS 100 controls 67 cases systemic lupus erythematosus	CSF-BACE CSF sAPPβ CSF sAPPα CSF Aβ Aβ_1–42_	Decreased BACE No differences in sAPPβ, sAPPα, Aβ
(33)	37 cases (RR) 10 controls	CSF sAPPβ CSF Aβ Aβ_1–40_ Aβ_1–42_	Decreased sAPPβ Decreased Aβ
(34)	42 cases (35 RR, 7 CIS, 5 PP) 12 controls	CSF Aβ Aβ_1–42_	Decreased Aβ
(35)	77 cases (42 MS, 10 NMO, 25 CIS) 21 controls	CSF sAPPα CSF Aβ Aβ_1–42_	No differences in sAPPα, Aβ
(27)	2 cases RR	PiB	Lower uptake in lesions in T1
(36)	65 cases (45 RR, 20 CIS) 83 controls	CSF Aβ	No differences in Aβ, although normal values were less frequent in RR patients than in controls.
(37)	74 cases (32 RR, 32 CIS, 10 PP) 74 controls	CSF Aβ Aβ_1–42_	No differences in Aβ
(38)	87 cases (54 RR, 33 SP) 28 controls	CSF sAPPα CSF sAPPβ CSF Aβ Aβ X_38_ Aβ X_40_ Aβ X_42_ Aβ_1–42_ Aβ peptides by mass spectrometry	Decreased sAPα Decreased sAPPβ Decreased Aβ
(28)	12 cases	PiB	Lower uptake in white matter lesions
(39)	14 cases (13 SP, 1 PP)	CSF anti-oligomer monoclonal antibodies	Detected
(29)	12 cases (5 RR, 5 SP, 2 PP)	^18^F-florbetaben	Lower uptake in white matter lesions than in normal-appearing white matter

**Table 2:** **MRI correlations with measurements related to the amyloid cascade in MS**.

Reference	Participants	Measure	MRI-related finding
(35)	77 cases (42 MS, 10 NMO, 25 CIS)	CSF sAPPα CSF Aβ_1–42_	No correlation with MRI atrophy
(34)	42 cases (35 RR, 7 CIS, 5 PP)	CSF Aβ_1–42_	Aβ levels were lower in Gd + MS patients No correlation was found with amyloid-β_1–42_ lesion load in T2 MRI sequences
(27)	2 cases (RR)	PiB-PET	Correlation between focal decreased amyloid uptake and T1 black holes
(29)	12 cases (5 RR, 5 SP, 2 PP)	^18^F-florbetaben	No correlation between uptake in white matter lesions and total lesion volume in T2 images

## Biomarkers of APP Proteolytic Processing in CSF in Patients with MS

Different studies evaluating Aβ levels in CSF in patients with clinically isolated syndrome (CIS) or MS have yielded divergent results (30, 31, 36, 37). However, it seems that levels of intermediate products of proteolysis of the amyloid precursor protein (APP), such as soluble α-APP and β-APP, and one of the final products, Aβ_1–42_, are reduced in patients with both the RR and the primary progressive forms of MS (34, 35, 38, 39). Likewise, there is an inverse correlation between Aβ levels and presence of gadolinium-enhancing lesions. Low activity of β-site APP-cleaving enzyme 1 (BACE1), the enzyme participating in amyloidogenic APP proteolysis, has also been demonstrated in CSF in patients with MS (32). However, these data are challenging to interpret, since CSF Aβ levels fluctuate throughout the day. This biomarker is therefore difficult to assess and extrapolating changes observed in CSF to demyelinating plaques is not always possible (Table 1). Altered Aβ CSF levels seem to be linked to situations of lower activity as shown by gadolinium uptake in MR images. These findings are not correlated with a greater degree of atrophy (Table 2).

## Effects of APP Proteolytic Processing in MS

In patients with MS, β-APP accumulates in damaged axons (40). Experimental allergic encephalomyelitis (EAE), an experimental model for MS, is more severe in association with a genetic deletion of APP. Pathology studies have found increased Aβ expression in demyelinating plaques (41–43), which may even provide protection from damage (44); in fact, treatment with either Aβ_42_ or Aβ_40_ reduces motor paralysis and brain inflammation and suppresses lymphocyte activation in animals with EAE. Similarly, decreased levels of pro-inflammatory cytokines and chemokines have been found in mice with EAE receiving Aβ peptides. Although these findings suggest that Aβ peptides are beneficial, we should not forget that they are neurotoxic and neuroinflammatory, and that APP proteolytic processing may provoke the opposite effect in demyelinated axons (45). This idea is consistent with studies describing increased Aβ_42_ levels in lesions and damaged axons. Several experimental studies report similar results: mice immunized with Aβ_1–42_ peptide experience symptoms whose presentation and pathological basis resemble those associated with EAE (46); Aβ injection in mice may damage the white matter (47) and induce oligodendrocyte death (48); and Aβ decreases the number of neurons in the subventricular zone and hippocampus and inhibits neurogenesis in the dentate gyrus of hippocampus, but not in the subventricular zone (49).

Amyloid precursor protein is extensively expressed in humans. Functions attributed to APP include neurite outgrowth and synaptogenesis, protein trafficking along axons, cell adhesion, calcium metabolism, and signal transduction (50). Due to the activity of several successive proteolytic processes involving α- and β-secretases (depending on whether the process is amyloidogenic), and subsequently γ-secretase, APP gives rise to soluble extracellular domains (sAPPα or sAPPβ) and the APP intracellular domain (AICD). Aβ is a protein with a great capacity to generate fibrils: it initially forms soluble monomers, and then oligomers, which remain soluble, until it ends up forming insoluble fibrils. Intracellular cascade of soluble peptides (β peptides, especially Aβ_40_ and Aβ_42_), which derive from APP proteolysis, may form oligomers and insoluble fibrillar deposits that become amyloid plaques (51). Another important fact is that APP is not an isolated protein, but rather one with two homologs: amyloid-like proteins 1 and 2, or APLP1 and APLP2 (52). Although genetic deletion of APP in mice provokes minor impairment (53), triple-knockout mice show such problems as perinatal death, cranial abnormalities, and cortical dysplasia (54, 55). The above suggests that APP family proteins fulfill essential yet partially redundant functions that can compensate for each other when several family members are present.

Although information on APP proteolytic processing in MS is scarce, we currently know that it is upregulated in damaged axons, which suggests that it may constitute a reliable marker of axon demyelination (56). Increased APP expression has been observed following compression injury in spinal cord white matter in rats (57). In APP knockout mice, nodal length is greater, and sodium channels are clustered. Spinal cord myelin sheaths are thinner in both APP knockout and APP-overexpressing transgenic mice (58). The potential impact of APP on MS may be related to coexpressing proteins. In fact, APP aggregates have been found in nodes of Ranvier, where APP expression colocalizes with tenascin-R, near the juxtaparanodal potassium channels. Tenascin-R is an extracellular matrix glycoprotein of the tenascin family that is exclusive to the CNS. It acts on cell differentiation, migration, and adhesion. Tenascin-R expression increases following microglial activation (59). It is upregulated by platelet-derived growth factor (PDGF) and participates in oligodendrocyte differentiation and consequently in remyelination (60). Tenascin-R has been studied in connection with MS due to its role in myelination (61), and expression has been shown to be reduced in chronic demyelinating plaques and present in acute and subacute plaques. Some studies therefore suggest that Tenascin-R inhibits remyelination (62) and prevents repair (63). APP has also been associated with Tau and αB-crystallin proteins in MS lesions, and αB-crystallin (HspB5) and Aβ peptides appear to be beneficial in EAE (64). A small heat-shock protein, αB-crystallin is highly immunogenic and associated with MS (65). It forms part of amyloid fibrils and improves EAE symptoms when administered systemically (66, 67). Other proteins that form part of amyloid fibrils are also beneficial, including Aβ A4, tau, amylin, and serum amyloid P (SAP). APP, αB-crystallin, and tau have been found in amyloid deposits in MS and they have demonstrated anti-inflammatory properties in MS animal models. The benefits of αB-crystallin are believed to be due to this protein’s ability to bind to pro-inflammatory proteins, and this ability increases in inflammatory processes. This activity takes place in a region of the molecule corresponding to the peptide that includes residues 73–92: in fact, this region alone is involved in EAE, and its activity is similar to that of the whole protein, which does not occur with other regions of the protein (68). This peptide can also form part of amyloid fibrils (69). At the same time, APP, αB-crystallin, SAP, and tau deficiencies in mice exacerbate EAE (70, 71). Furthermore, administration of the hexapeptide complex comprising the proteins included in amyloid fibrils rapidly decreases plasma levels of such pro-inflammatory cytokines as IL-6 and IL-2 (72).

Another relevant enzyme is BACE1, a membrane-bound aspartyl protease (73). It is the only enzyme that directly breaks down APP to generate Aβ (74), and it accumulates in AD brains (75–79). BACE1-knockout mice also lack Aβ (80–82). Genetic deletion of BACE1 during development leads to hypomyelination in the central and peripheral nervous systems (83, 84), and the enzyme is necessary for sciatic nerve remyelination after an injury (85). The role of BACE1 in myelination may be explained by the fact that it processes neuregulin-1 and -3 (NRG1, NRG3) (86). Members of the NRG family of proteins are neurotrophic factors that act on ErbB receptors and trigger a biochemical cascade regulating several functions, including myelination. Decreased activity in this signaling pathway reduces myelin sheath thickness (87–90). This suggests that β secretase may play a crucial role in remyelination in MS.

On the other hand, Aβ peptides can trigger microglial activation (91–93). Microglial activation induced by Aβ *in vivo* is accompanied by decreased CD200 neuronal expression. The CD200 protein controls microglia and assists in inflammatory processes (94, 95).

## Proteins Involved in APP Proteolytic Processing in Demyelination

The role of APP and its homologs in demyelination may be due to APP proteolytic processing via substrates and enzymes. Both β- and γ-secretase are located in the lipid raft of the cell membrane, which contains sphingolipids and cholesterol (96). This lipid composition of the membrane influences β- and γ-secretase activity (97–99). The potential role of lipid components in APP proteolytic processing has been extensively reviewed (100); Aβ production is modulated by sphingolipids. Demyelination leads to a release of myelin proteins (101): Nogo, myelin-associated glycoprotein, and oligodendrocyte myelin glycoprotein inhibit neuronal regeneration via Nogo and PirB receptors (102, 103), and MBP causes damage since it acts directly on the neuronal membrane (104). This protein, which has been regarded as one of the antigens for MS, performs many functions: it is involved in Aβ aggregation and inhibits Aβ fibril assembly (105), which affects Aβ levels. In experimental models, brain tissue inflammation followed by ischemia produces axonal and myelin damage with myelin aggregates that colocalize with APP and Aβ. In the 5XFAD mouse model, Aβ plaques were observed to colocalize with myelin aggregates (106). As shown by *in vitro* studies, MBP inhibits Aβ fibril assembly via residues 1–64 (107), a fragment known as MBP1 (108). MBP1 has been proven to reduce pathological Aβ accumulation and clinical alterations in the 5XFAD mouse (109). This occurs in control animal models and has also been observed in models presenting mutant forms of Aβ (Dutch- and Iowa-type Aβ) that are responsible for cerebral amyloid angiopathy, in which MBP inhibits fibril formation (105). Although MBP1 may have a protective role in AD, it may be harmful in MS since it reduces amyloid fibril production, which favors the detrimental effect of Aβ peptides.

## Conclusion

Tracer uptake in white matter in amyloid PET imaging studies has raised questions about its utility as a biomarker of demyelination, specifically in white matter diseases such as MS. Several studies have aimed to determine how remyelination and MS are affected by APP and the proteins expressed via APP proteolytic processing, and whether amyloid-PET can provide an *in vivo* molecular diagnosis of this process. Although further research on APP in MS is necessary, recent studies have demonstrated that (1) APP does play a role in MS; (2) APP proteolytic processing occurs as a result of demyelination, due to the action of myelin protein or lipid detritus; and (3) APP is involved in remyelination to a greater or lesser extent. In conclusion, amyloid-PET may serve as a tool for determining the degree of demyelination and remyelination as well as a means of studying molecular changes linked to remyelination in MS *in vivo*.

## Author Contributions

All authors listed, have made substantial, direct, and intellectual contribution to the work, and approved it for publication.

## Conflict of Interest Statement

The authors declare that the research was conducted in the absence of any commercial or financial relationships that could be construed as a potential conflict of interest. The reviewers, CE and RD, and the handling Editor, declared their shared affiliation, and the handling Editor states that the process nevertheless met the standards of a fair and objective review.
